# Influence of growth temperature on thermal tolerance of leading foodborne pathogens

**DOI:** 10.1002/fsn3.1268

**Published:** 2019-11-16

**Authors:** Chyer Kim, Rana Alrefaei, Mariam Bushlaibi, Eunice Ndegwa, Paul Kaseloo, Crystal Wynn

**Affiliations:** ^1^ Agricultural Research Station Virginia State University Petersburg VA USA; ^2^ Department of Biology Virginia State University Petersburg VA USA; ^3^ Department of Family and Consumer Sciences Virginia State University Petersburg VA USA

**Keywords:** *D‐*value, foodborne pathogens, incubation temperature, thermal destruction rate, *z‐*value

## Abstract

Accurate prediction of the thermal destruction rate of foodborne pathogens is important for food processors to ensure proper food safety. When bacteria are subjected to thermal stress during storage, sublethal stresses and/or thermal acclimation may lead to differences in their subsequent tolerance to thermal treatment. The aim of the current study was to evaluate the thermal tolerance of *Escherichia coli* O157:H7, *Listeria monocytogenes*, *Salmonella enterica*, and *Staphylococcus aureus* that are incubated during overnight growth in tryptic soy broth at four temperatures (15, 25, 35, and 45°C). Following incubation, the bacteria were subjected to thermal treatments at 55, 60, and 65°C. At the end of each treatment time, bacterial survival was quantified and further calculated for the thermal death decimal reduction time (*D‐*value) and thermal destruction temperature (*z‐*value) using a linear model for thermal treatment time (min) vs. microbial population (Log CFU/ml) and thermal treatment temperature (°C) vs. *D‐*value, respectively, for each bacterium. Among the four bacterial species, *E. coli* generally had longer *D‐*values and lower *z‐*values than did other bacteria. Increasing patterns of *D‐* and *z‐*values in *Listeria* were obtained with the increment of incubation temperatures from 15 to 45°C. The *z‐*values of *Staphylococcus* (6.19°C), *Salmonella* (6.73°C), *Listeria* (7.10°C), and *Listeria* (7.26°C) were the highest at 15, 25, 35, and 45°C, respectively. Although further research is needed to validate the findings on food matrix, findings in this study clearly affirm that adaptation of bacteria to certain stresses may reduce the effectiveness of preservation hurdles applied during later stages of food processing and storage.

## Graphical abstract

When bacteria are subjected to thermal shock during storage, sublethal stresses and/or thermal acclimation may lead to differences in their subsequent tolerance to thermal treatment. We found that increasing pattern of bacterial thermal tolerance was observed with the increment of thermal stress. Although further research is needed to validate the current findings on food matrices, findings in this study clearly affirm that adaptation of bacteria to certain stresses may reduce the effectiveness of preservation procedures applied during later stage of food processing and storage.

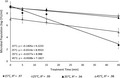

## 1 INTRODUCTION

During a thermal process, the rate of bacterial destruction is dependent both on the temperature of exposure and on the time maintained at this temperature (Anonymous, 2017). Thermal destruction rates are mostly displayed with thermal death decimal reduction time (*D‐*value) and thermal death decimal reduction temperature (*z‐*value), and each species of bacteria have its own particular heat tolerance. Therefore, it is essential to determine the *D‐* and *z‐*values of each bacterium to identify how much heat is required to destroy a given bacterium. The *D‐*value is defined as the time required at a constant temperature to destroy 90% of the bacteria present, while a *z‐*value is defined as the change in temperature necessary to bring about the 90% change in *D‐*value, which indicates shortening the duration of time with the increase of heat. Hence, they can be used as predictors for how susceptible a bacteria is to changes in temperature and in duration.

When bacteria are subjected to thermal stress during food processing treatments, sublethal stresses and thermal acclimation can occur that may lead to differences in terms of their tolerance to the heat process that follows. Furthermore, most research documenting the time and temperature relationship necessary for the destruction of bacteria has been conducted using a limited number of different bacterial strains or species in isolation (Patchett, Watson, Fernández, & Kroll, 1996; Redondo‐Solano, Burson, & Thippareddi, 2016; Semanchek & Golden, 1998; Sörqvist, 2003). These studies are difficult to compare due to differences in multiple variables, including between strains or species of bacteria, between research laboratories and environmental conditions, and between technologies used for the evaluation of microbial destruction. Therefore, culturing and evaluating bacterial pathogens under concurrent conditions will better elucidate the net effect of growth temperature on inactivation and injury of pathogens due to thermal process and thereby facilitate cross‐species comparisons.

*Escherichia coli* O157:H7, *Listeria monocytogenes, Salmonella enterica*, and *Staphylococcus aureus* are the leading bacteria accountable for the vast majority of foodborne illnesses, hospitalizations, and deaths in the United States (CDC 2016). Recognizing the importance of these pathogens associated with various food products, as a preliminary phase, the objective of current study was to evaluate the thermal tolerance (*D‐* and *z‐*values) of different species of bacteria grown in a laboratory culture medium (tryptic soy broth supplemented with 0.6% yeast extracts) at four different temperatures (15, 25, 35, and 45°C). In addition, the term “cool,” “ambient,” “warm,” and “excessive heat” for 15, 25, 35, and 45°C, respectively, defined as in U.S. Pharmacopeia 659 (USP 2017) are used for description purpose of thermal stresses in this article.

## 2 MATERIALS AND METHODS

### 2.1 Bacterial strains used

Bacterial species used for the study were obtained from American Type Culture Collection (ATCC, Manassas, VA). In brief, three strains of *Escherichia coli* O157:H7 (35,150, human feces isolate; 43,888, human feces isolate; and 700,728, quality control strain), four strains of *Listeria monocytogenes* (7,644, human isolate; 19,115, human isolate; 43,256, cheese isolate; and 51,772, dairy product isolate), four serovars of *Salmonella enterica* (Enteritidis, emerging infectious disease research strain; Montevideo, emerging infectious disease research strain; Newport, food poisoning isolate; and Typhimurium, chicken isolate), and four strains of *Staphylococcus aureus* (6,538, human lesion isolate; 29,213, human wound isolate; 33,862, enteric research strain; and 49,444, dairy product isolate) were used. Stock cultures of each pathogen strain were maintained in tryptic soy broth (TSB; pH 7.4, unless otherwise stated, all media were Bacto, from Becton Dickinson) containing 20% (vol/vol) glycerol (Thermo Scientific) and kept frozen at −80°C. Cultures were transferred three times to TSB supplemented with 0.6% yeast extract (TSBYE) by loop inoculation at successive 24‐hr intervals and incubated at 35°C before they were used for the study.

### 2.2 Growth temperature

In order to investigate thermal destruction variability in foodborne pathogens induced by thermal stresses during storage in optimum medium, 0.1 ml of each strain was inoculated into 10 ml TSBYE and incubated for 24 hr at 15, 25, 35, and 45°C. Following incubation, the bacteria were centrifuged for 10 min at 2,000 *g* and 22 ± 2°C in a centrifuge (Model Heraeus Megafuge 16, Thermo Scientific). The pellets were then suspended in 10 ml of sterile 0.85% saline solution and centrifuged again at 2,000 *g* for 10 min and resuspended in 10 ml of sterile 0.85% saline solution. Equal volumes of either three or four strains of each bacterial species were combined to make an inoculum containing approximately equal numbers of cells of each species of *E. coli* O157:H7, *L. monocytogenes*, *S. enterica*, or *S. aureus*. In other words, a cocktail containing either 3 or 4 strains of each bacterial species was used as inocula for thermal destruction rate study. Levels of bacterial counts obtained after 24‐hr incubation at each temperature are shown in Table 1 and used as inocula for the thermal destruction study. The inoculum levels shown in Table 1 are the average of three independent replicate trials of each bacterial population prior to being subjected to thermal treatments at 55, 60, and 65°C. Three independent replicate trials were conducted for each thermal treatment at each incubation temperature (thermal stress) of 15, 25, 35, and 45°C. Species identity was periodically confirmed on eosin methylene blue agar (EMB) for *E. coli* O157:H7, modified oxford agar (MOX) for *L. monocytogenes*, Baird‐Parker agar supplemented with egg yolk tellurite (BP) for *S. aureus*, and xylose lysine deoxycholate agar (XLD) for *S. enterica*. In addition, AOAC‐approved or performance‐tested methods (TECRA, 2004, 2005) including API 20E, API Listeria, and rabbit plasma test were performed.

**Table 1:** The level of bacterial species obtained after 20 ± 2‐hr incubation at various temperatures and used as inocula

Bacterial species	Inoculum level (Log CFU/ml)/incubation temperature (°C).			
	15	25	35	45
*E. coli* O157:H7	8.47 ± 1.06Aa	8.93 ± 0.11Aa	8.92 ± 0.10Aa	6.80 ± 0.32ABb
*L. monocytogenes*	7.97 ± 0.22Aa	8.86 ± 0.25Aa	8.77 ± 0.01Aa	3.54 ± 1.22Cb
*S. enterica*	8.42 ± 0.07Aa	9.07 ± 0.03Aa	8.13 ± 1.58Aa	8.03 ± 0.14Aa
*S. aureus*	6.87 ± 0.05Bb	9.14 ± 0.11Aa	9.07 ± 0.00Aa	5.67 ± 0.52Bc

Note

Means followed by the same uppercase letters in the same column are not significantly different (*p* > .05); means followed by the same lowercase letters in the same row are not significantly different (*p* > .05); data are expressed as means ± standard error (*n* = 3).

### 2.3 Thermal destruction

Each bacterial species of inoculum (2 ml) was introduced into a sterile polyethylene whirl‐pak® sample bag (Nasco) and sealed. The bags were 7.5 × 12.5 cm in size with a thickness of 0.057 mm. The sample bags were then completely immersed in a water bath (Lab‐Line Water Bath Model 18900 AQ, Thermo Scientific) and held at 55°C for 300, 900, and 2,700 s; 60°C for 30, 90, 270 s; and 65°C for 3, 9, and 27 s. These ranges of temperatures, which are commonly used in cooking beef up to medium‐rare, were chosen in this study for future validation study in mind on food matrices such as ground beef and roasted beef (Line et al., 1991). The sample temperature was monitored using a thermocouple connected to a thermometer (Traceable Infrared Dual‐Lasers Thermometer, Model S02273, Control Co.). At the end of each exposure time, the sample bags were removed from the water bath and immediately immersed in ice water (0°C) for 5 min to stop further inactivation due to thermal treatment. Bacterial suspensions in the sample bags were then serially diluted in sterile 0.85% saline solution, surface‐plated on standard method agar (SMA), and incubated at 35°C for 48 hr prior to quantification of bacterial survival. The counts were expressed as log colony‐forming unit (CFU)/ml.

### 2.4 Calculation of D‐ and z‐values

**Figure 1:**
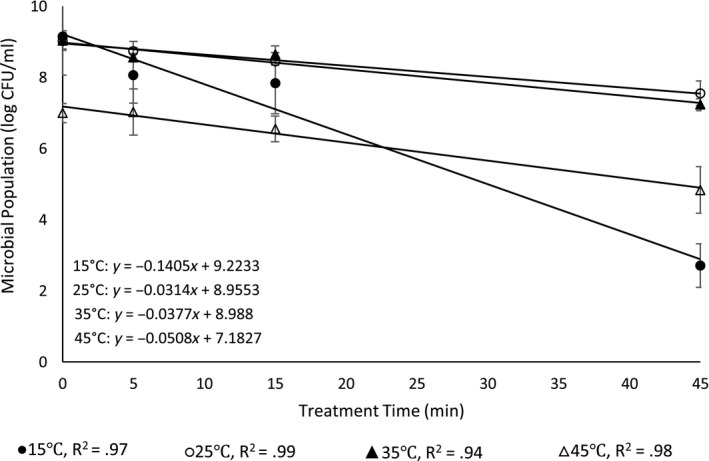
Representative thermal inactivation curves of *E. coli* that incubated for 24 hr in TSBYE at 15 (close circles), 25 (open circles), 35 (close triangles), and 45°C (open triangles) and subsequentially subjected to thermal treatment at 55°C. Thermal inactivation gradients are shown in trend line equations. Data are expressed as means ± standard error (*n* = 3)

The destruction rate curves (*R* ^2^ ≥ .89) were constructed by plotting the bacterial survivors on the logarithmic scale against the respective exposure time on the linear scale. As described in Redondo‐Solano et al. (2016), the slopes of the thermal destruction rate curves in decimal reduction times (*D‐*values) for species of *E. coli* O157:H7, *L. monocytogenes*, *S. enterica*, and *S. aureus* were calculated from linear regression (inverse of the slope of the regression line) using Excel software (2013, Microsoft) and expressed in minutes. A representative example of the linear regression fitted thermal destruction curves obtained from the bacteria (*E. coli* O157:H7) that subjected to thermal treatment at 55°C following 24‐hr incubation at 15, 25, 35, and 45°C is shown in Figure 1. The thermal destruction temperature (*z‐*values) was also calculated by plotting the temperature against log *D‐*value, and the data were fitted by using linear regression with Excel software (2013, Microsoft). The inverse of the slope was reported as the *z‐*value in °C.

### 2.5 Statistical analysis

The thermal destruction times (*D‐*values) and temperatures (*z‐*values) for species of *E. coli* O157:H7, *L. monocytogenes*, *S. enterica*, and *S. aureus* were obtained from three independent replications. Data (log CFU/ml, *D‐*values, and *z‐*values) were subjected to an analysis of variance and Duncan's multiple range test (SAS Institute) to determine the significance of the differences (*p* < .05) in mean values.

## 3 RESULTS AND DISCUSSION

The effect of thermal stresses (incubation temperature) on the level of bacteria in TSBYE after 20 ± 2‐hr incubation is shown in Table 1. The levels of bacteria subjected to the highest incubation temperature (45°C) were the lowest for all bacterial species. *Salmonella* only was not significantly (*p* > .05) affected by thermal stresses. The level of *Staphylococcus* only incubated at 15°C was significantly (*p* < .05) lower than those incubated at 25 and 35°C indicating that *Staphylococcus* species were more susceptible to cool temperature stress than other bacterial species. It was also noted that *Staphylococcus* was more susceptible to heat than cold stress resulting in significantly lower level at 45°C than 15°C after 24‐hr incubation. After 24‐hr incubation at 45°C, the level of *Salmonella* was the highest while the level of *Listeria* was the lowest among tested bacterial species indicating that *Salmonella* was the least susceptible and *Listeria* was the most susceptible to thermal stresses at 45°C. Overall, all tested bacterial species except *Staphylococcus* were able to maintain optimum populations at temperatures from 15 to 35°C after 20 ± 2‐hr incubation showing insignificant (*p* > .05) differences in‐between.

### 3.1 *E. coli* O157:H7

The inoculum level of *E. coli* (Table 1) incubated at 15, 25, 35, and 45°C decreased by 6.44 ± 1.67, 1.47 ± 0.11, 1.80 ± 0.18, and 2.16 ± 0.43 log CFU/ml, respectively, after 45 min of thermal treatment at 55°C (Figure 1). In addition, the thermal inactivation gradient (the magnitude of bacterial population reduction represented as a slope of linear regression, log CFU vs. thermal treatment time) of *E. coli* incubated at 15°C (−0.1405) was significantly (*p* < .05) higher than those incubated at 45°C (−0.0508), 35°C (−0.0377), and 25°C (−0.0314) in decreasing order. Similar observations were also made for the bacteria that subjected to thermal treatment at 60 and 65°C for 270 and 27 s, respectively, (data not shown) indicating that the bacteria grown at 15°C were the most susceptible to thermal treatments among the tested incubation temperatures.

The *D‐*values (microbial population vs. treatment time) and *z‐*values (log *D‐*value vs. treatment temperature) calculated by fitting the primary log‐linear model to the thermal inactivation curves are shown in Table 2. When the *D‐*values were evaluated at 55°C, the bacteria incubated at 15°C were the shortest (7.2 ± 0.73 min), followed by 45°C (19.9 ± 2.74 min), 35°C (25.77 ± 1.96 min), and 25°C (31.9 ± 2.63 min) indicating that the bacteria subjected to cool temperature (15°C) and excessive heat temperature (45°C) stress were more susceptible to the thermal treatment at 55°C than those subjected to ambient (25°C) and warm (35°C) temperatures. Table 2 also revealed that it took a longer time to reduce the level of the bacteria incubated at 25°C by 90% at 55°C thermal treatment compared with the bacteria incubated at 35°C, which is closer to the optimum temperature (37°C, Albrecht, 2017) for bacterial growth. While the *D‐*value of the bacteria incubated at 15°C was the shortest (0.62 ± 0.08 min) at thermal treatment of 60°C, similar to the observation made at 55°C, there were no significant differences of *D‐*values among other incubation temperatures (25, 35, and 45°C). However, at 65°C thermal treatment, the bacteria incubated at 35°C showed the longest *D‐*value (0.61 ± 0.16 min) and significantly (*p* < .05) longer than those incubated at 15, 25, and 45°C. This indicates that the bacteria subjected to warm temperature (35°C) stress were more resistant to the highest thermal treatment (65°C) in this study.

**Table 2:** *D*‐(min) and *z*‐values (°C) of *E. coli* O157:H7 that incubated during overnight growth (20 ± 2 hr) at four temperatures (15, 25, 35, and 45°C) and subsequentially subjected to thermal treatments at 55, 60, and 65°C in 0.85% saline solution

Incubation temperature (°C)	Thermal treatment (°C)	*D*‐value	*R* ^2^ for *D*‐value	*z*‐value	*R* ^2^ for *z*‐value
15	55	7.21 ± 0.98Ad	.97	5.03 ± 0.12b	.99
	60	0.62 ± 0.08Bb	.95		
	65	0.07 ± 0.01Bb	.94		
25	55	31.91 ± 2.63Aa	.99	4.27 ± 0.49c	.99
	60	1.36 ± 0.33Ba	.98		
	65	0.15 ± 0.08Bb	.95		
35	55	25.77 ± 1.97Ab	.94	6.13 ± 0.32a	.97
	60	1.20 ± 0.23Ba	.99		
	65	0.61 ± 0.16Ba	.99		
45	55	19.91 ± 2.75Ac	.98	4.67 ± 0.38bc	.99
	60	1.61 ± 0.41Ba	.98		
	65	0.14 ± 0.04Bb	.98		

Note

Means followed by the same uppercase letters in the same column within the same incubation temperature are not significantly different (p > .05); means followed by the same lowercase letters in the same column within the same thermal treatment temperature are not significantly different (p > .05); z‐values followed by the same lowercase letters are not significantly different (p > .05); data are expressed as means ± standard error (n = 3).

Findings from our study agree with the results presented by Jackson, Hardin, and Acuff (1996) that stationary‐phase populations of *E. coli* O157:H7 grown at 37°C were more resistant to heat treatment than populations grown at 23 and 30°C. Studies have also indicated that heat tolerance was greater when cells were grown at 37 or 40°C rather than 10, 23, 25, or 30°C (Jackson et al., 1996; Kaur, Ledward, Park, & Robson, 1998; Semanchek & Golden, 1998). Katsui, Tsuchido, Takano, and Shibasaki (1981) reported that increased heat tolerance associated with changes in growth temperature were attributable to alteration of the fatty acid composition in bacterial membranes. Another supporting study done by Beuchat (1978) indicated that bacteria grown at low temperatures may incorporate more unsaturated fatty acids into their cell membranes in order to maintain functional membrane fluidity. Therefore, decreased heat tolerance may occur due to the reduced melting point of unsaturated fatty acids within the cell membrane. In addition, scientists have reported that the stress response of *E. coli* O157:H7 to sublethal environmental stresses such as changes in temperature, starvation, or high osmolarity provides cross‐protection to a variety of postphysical and postchemical stresses including heat and acid (Allen, Lepp, McKellar, & Griffiths, 2010; Arnold & Kaspar, 1995; House et al., 2009; Jeong, Baumler, & Kaspar, 2006; Lange & Hengge‐Aronis, 1994; Leenanon & Drake, 2001; Nair & Finkel, 2004).

In general, *E. coli* subjected to thermal treatment at 55°C showed the highest heat tolerance (the longest *D‐*value) compared with those subjected to 60 and 65°C. The length of time required to destroy 90% of the bacteria cultured/grown at 15°C was the shortest among those cultured at 25, 35, and 45°C. The increase (*z‐*value, 6.13 ± 0.32°C) in temperature needed to bring about the 90% reduction in the duration of treatment time (*D‐*value) was the highest for the bacteria incubated at 35°C, and the difference was significant (*p* < .05). Findings in this study clearly demonstrated the difference in *E. coli* O157:H7 thermal tolerance due to their prior thermal stresses (incubation temperatures).

### 3.2 L. monocytogenes

The reduction level of *Listeria* inoculum (Table 1), which was incubated at 15°C and subsequentially subjected to thermal treatments at 55, 60, and 65°C, was the highest among the tested incubation temperatures (Data not shown). Thermal reduction gradients of bacteria incubated at 15°C were the highest and the lowest at 45°C indicating that the bacteria grown at 15 and 45°C were the most and least susceptible, respectively, to thermal treatments. Results shown in Table 3 also reveal that the *D‐*values obtained from the bacteria incubated at 15 and 45°C were the shortest and the longest, respectively. This confirmed that the bacteria subjected to cool temperature (15°C) and excessive heat temperature (45°C) stress were the most and least susceptible, respectively, to each thermal treatment at 55, 60, and 65°C. In other words, it required less time for the bacteria incubated at lower temperature to be reduced by 90% as those subjected to higher incubation temperatures. However, at 60°C thermal treatment, the bacteria subjected to ambient temperature (25°C) stress demonstrated longer *D‐*value than those subjected to warm temperature (35°C) stress, which was contradictory to the overall increasing trends of *D‐*values with increasing incubation temperatures as shown in Table 3.

**Table 3:** *D*‐(min) and *z*‐values (°C) of *L. monocytogenes* that incubated during overnight growth (20 ± 2 hr) at four temperatures (15, 25, 35, and 45°C) and subsequentially subjected to thermal treatment at 55, 60, and 65°C in 0.85% saline solution

Incubation temperature (°C)	Thermal treatment (°C)	*D*‐value	*R* ^2^ for *D*‐value	*z*‐value	*R* ^2^ for *z*‐value
15	55	8.50 ± 0.10Ac	.97	5.29 ± 0.21b	.96
	60	0.61 ± 0.03 Bd	.98		
	65	0.11 ± 0.02Cb	.98		
25	55	10.94 ± 0.61Ab	.98	5.38 ± 1.02b	.99
	60	1.18 ± 0.08Bb	.99		
	65	0.17 ± 0.11Cb	.99		
35	55	11.63 ± 0.23Aab	.99	7.10 ± 0.20a	.88
	60	0.79 ± 0.12Bc	.99		
	65	0.45 ± 0.04Ca	.97		
45	55	12.39 ± 0.54Aa	.96	7.26 ± 1.20a	.96
	60	1.84 ± 0.10Ba	.90		
	65	0.67 ± 0.26Ca	.99		

Note

Means followed by the same uppercase letters in the same column within the same incubation temperature are not significantly different (p > .05); means followed by the same lowercase letters in the same column within the same thermal treatment temperature are not significantly different (p > .05); z‐values followed by the same lowercase letters are not significantly different (p > .05); data are expressed as means ± standard error (n = 3).

A review (Sörqvist, 2003) of thermal tolerance of *L. monocytogenes* grown in either conventional media, milk, or liquid product at 30–37°C indicated that although various methods of heat treatment were applied (e.g., heating in water baths using glass capillary tubes, sealed glass tubes, glass ampoules or polyethylene pouches completely immersed in the water), all means of *D‐*values obtained at 55, 60, and 65°C were 10.71, 1.45, and 0.2 min, respectively, which agree reasonably well with the results found in our study (10.94–11.63 min at 55°C, 0.79–1.18 min at 60°C, and 0.17–0.45 min at 65°C) on *L. monocytogenes* grown at 25 and 35°C (Table 3). Additionally, studies (Mackey & Bratchell, 1989; Skandamist et al. 2008) reported the adaptive thermotolerance responses on *L. monocytogenes* that subjected to prior thermal stresses.

In general, *D‐*value of *Listeria* incubated at 15 and 45°C was the shortest and the longest, respectively. *z‐*value (7.26 ± 1.20°C) was the highest for the bacteria incubated at the highest temperature (45°C). Increasing pattern of *z‐*value in *Listeria* was obtained with the increment of incubation temperatures from 15 to 45°C in sequential order.

### 3.3 Salmonella

Reduction gradients of the bacteria incubated at 25°C and 45°C were the highest and the lowest, respectively, when they were subsequentially subjected to thermal treatments at 55 and 60°C (Data not shown). However, at 65°C thermal treatment, the reduction gradient for the bacteria incubated at 15°C and 35°C was the highest and the lowest, respectively. Results in Table 4 displayed that *D‐*values obtained from the bacteria incubated at 45°C were the longest indicating that the bacteria subjected to the highest thermal stress (excessive heat temperature, 45°C) were the least susceptible to subsequential thermal treatments at each 55, 60, and 65°C. It was also noted that *D‐*values* of* the bacteria subjected to subsequential thermal treatment at 60°C were not significantly (*p* > .05) affected by prior thermal stresses from 15 to 35°C.

**Table 4:** *D*‐(min) and *z*‐values (°C) of *S. enterica* that incubated during overnight growth (20 ± 2 hr) at four temperatures (15, 25, 35, and 45°C) and subsequentially subjected to thermal treatment at 55, 60, and 65°C in 0.85% saline solution

Incubation temperature (°C)	Thermal treatment (°C)	*D*‐value	*R* ^2^ for *D*‐value	*z*‐value	*R* ^2^ for *z*‐value
15	55	5.82 ± 0.24Ac	.95	5.85 ± 0.56bc	.99
	60	0.86 ± 0.12Bb	.95		
	65	0.12 ± 0.05Cb	.96		
25	55	5.29 ± 0.71Ac	.98	6.73 ± 0.28a	.97
	60	0.70 ± 0.14Bb	.95		
	65	0.16 ± 0.02Bb	.99		
35	55	8.71 ± 0.79Ab	.98	6.64 ± 0.55ab	.97
	60	0.94 ± 0.11Bb	.93		
	65	0.27 ± 0.06Ba	.99		
45	55	18.59 ± 0.87Aa	.95	5.47 ± 0.31c	.98
	60	1.47 ± 0.17 B a	.98		
	65	0.28 ± 0.06Ca	.98		

Note

Means followed by the same uppercase letters in the same column within the same incubation temperature are not significantly different (p > .05); means followed by the same lowercase letters in the same column within the same thermal treatment temperature are not significantly different (p > .05); z‐values followed by the same lowercase letters are not significantly different (p > .05; data are expressed as means ± standard error (n = 3).

Thermal tolerance study (Amado, Vázquez, Guerra, & Pastrana, 2014) in phosphate‐buffered saline (PBS) solution on *Salmonella enterica*, which were isolated from vegetable feed ingredients and grown at 37°C, showed *D‐*values of 0.44–1.35 min at 60°C and 0.22–0.66 min at 65°C, which agree well with the results found in our study (0.94 and 0.27 min at 60 and 65°C, respectively) on *Salmonella* grown at 35°C. Another study done on *Salmonella Bedford* strain by Baird‐Parker, Boothroyd, and Jones (1970) obtained *D‐*values of 18.8 and 4.3 min at 55 and 60°C, respectively, while *D‐*value results found in our study on *Salmonella enterica* incubated at 15 to 45°C ranged from 5.29–18.59 min at 55°C to 0.70–1.47 min at 60°C. These differences may be due to the difference in the tested strains and the study being conducted in broth without subjecting cells to washing process. Additionally, different survival/growth rates at different incubation temperatures (Table 1) may have contributed to these results. Therefore, further research is in consideration to manifest the influence of bacterial growth phase on their thermal resistance.

In general, the time required to destroy 90% of *Salmonella* grown at 45°C was the longest compared with those incubated at 15, 25, and 35°C. The increase in temperature needed to bring about the 90% reduction in the duration of time (*D‐*value) was the highest and lowest for *Salmonella* grown at 25 (6.73 ± 0.28°C) and 45°C (5.47 ± 0.31°C), respectively. The bacteria grown at lower temperature (25°C) required shorter time but higher temperature increase to be destroyed than the bacteria grown at higher temperature (45°C) indicating that destruction of *Salmonella* bacteria grown at low and high temperature may be more dependent upon duration of time and increase of temperature, respectively.

### 3.4 Staphylococcus

The reduction level of *Staphylococcus* inoculum incubated at 45°C and subsequentially subjected to thermal treatments at 55, 60, and 65°C was the highest among the four tested incubation temperatures (Data not shown). At 55 and 60°C thermal treatments, reduction gradients of the bacteria incubated at 45°C were the highest and the lowest at 35°C. However, at 65°C thermal treatment, the reduction gradient was the highest and the lowest at 35°C and 25°C, respectively. This discrepancy of the bacterial response to thermal treatments clearly demonstrates thermal tolerance dissimilarity of the bacteria due to prior thermal stresses as well as thermal treatment temperatures. Results in Table 5 demonstrated that the bacteria incubated at 35°C, which is close to the optimum temperature (37°C, Albrecht, 2017; NZMPI 2001) for *Staphylococcus* growth, took the longest time (*D‐*value) to reduce its population by 90%. While *D‐*value of the bacteria incubated at 45°C was significantly (*p* < .05) lower at thermal treatment of 55°C than those incubated at other temperatures, no significant difference of *D‐*values among the bacteria that subjected to other incubation temperatures (15, 25, and 35°C) and thermal treatments (60 and 65°C) was observed. However, the increase in temperature (*z‐*value, 6.77 ± 0.52°C) needed to bring about the 90% reduction in the duration of time (*D‐*value) was the highest for the bacteria incubated at the highest incubation temperature (45°C).

**Table 5:** *D*‐(min) and *z*‐values (°C) of *S. aureus* that incubated during overnight growth (20 ± 2 hr) at four temperatures (15, 25, 35, and 45°C) and subsequentially subjected to thermal treatment at 55, 60, and 65°C in 0.85% saline solution

Incubation temperature (°C)	Thermal treatment (°C)	*D*‐value	*R* ^2^ for *D*‐value	*z*‐value	*R* ^2^ for *z*‐value
15	55	11.23 ± 1.10Aa	.96	6.19 ± 0.38ab	.99
	60	1.41 ± 0.02Ba	.97		
	65	0.31 ± 0.06Ba	.89		
25	55	12.04 ± 1.66Aa	.99	6.14 ± 0.79ab	.99
	60	1.51 ± 0.28Ba	.99		
	65	0.35 ± 0.13Ba	.97		
35	55	13.42 ± 0.79Aa	.97	5.63 ± 0.29b	0.99
	60	1.57 ± 0.25Ba	.95		
	65	0.22 ± 0.04Ca	.96		
45	55	9.45 ± 0.43Ab	.96	6.77 ± 0.52a	.99
	60	1.36 ± 0.19Ba	.89		
	65	0.32 ± 0.07Ba	.98		

Note

Means followed by the same uppercase letters in the same column within the same incubation temperature are not significantly different (p > .05); means followed by the same lowercase letters in the same column within the same thermal treatment temperature are not significantly different (p > .05); z‐values followed by the same lowercase letters are not significantly different (p > .05); data are expressed as means ± standard error (n = 3).

In general, the *D‐*values (1.57 min at 60°C and 0.22 min at 65°C) obtained from our study on the bacteria incubated at 35°C were similar to those previously reported by Amado et al. (2014). In their study, the average *D‐*values of seven *Staphylococcus* isolates grown at 37°C and tested in PBS solution were 1.35 min at 60°C and 0.37 min at 65°C. However, at 55°C, higher thermal tolerance was observed in the isolates (13.42 min) assayed in our study compared with theirs (3.82 min). Amado et al. (2014) indicated that *D‐*values obtained in their study were lower than those (Kennedy, Blair, McDowell, & Bolton, 2005) reported for *Staphylococcus*. Kennedy et al. (2005) reported *D‐*values at 55°C ranging from 13.7 to 21 min, which were much more similar to our findings. As described by Jung and Beuchat (2000), discrepancies in *D‐*values reported in our study compared with those described in Amdao et al. (2014) can be due to variations in experimental conditions, in terms of both strain and medium in which the thermal tolerance is studied (i.e., TSB, phosphate‐buffered saline solution [PBS], peptone water), conditions of microbial growth, etc. Oh et al. (2009) and Franz, Hoek, Bouw, and Aarts (2011) reported that the survival capabilities of bacteria also depend on isolate source and type of environmental stress.

For the statistical comparison purpose of thermal tolerance across species, Tables 2, 3, 4, 5 are compiled and summarized in Table 6. At cool temperature (15°C) stress, *Staphylococcus* displayed the longest *D‐*value with significant difference (*p* < .05) at all tested thermal treatment temperatures. *Salmonella* (5.82 min), *Listeria* (0.61 min), and *E. coli* (0.07 min) demonstrated the shortest *D‐*values at 55, 60, and 65°C, respectively. The increase in temperature needed to bring about the 90% reduction in the duration of time was the highest (6.19°C) for *Staphylococcus* and the lowest (5.03°C) for *E. coli*, reaffirming the results obtained from *D‐*values that *Staphylococcus* was the most thermal tolerant at 15°C incubation. At ambient temperature (25°C) stress, *E. coli* demonstrated the longest *D‐*value at 55°C (31.9 min), while *Staphylococcus* showed the longest *D‐*values at 60°C (1.51 min) and 65°C (0.35 min). *Salmonella* demonstrated the shortest *D‐*values at 55°C (5.29 min) and 60°C (0.70 min). At warm temperature (35°C) stress, *E. coli* had the longest *D‐*values at both 55°C (25.77 min) and 65°C (0.61 min), and *Staphylococcus* at 60°C (1.57 min). *Salmonella*, *Listeria*, and *Staphylococcus* had the lowest *D‐*value at 55°C (8.71 min), 60°C (0.79 min), and 65°C (0.22 min), respectively. The *z‐*value was the highest for *Listeria* (7.10°C) and the lowest for *Staphylococcus* (5.63°C). At excessive heat temperature (45°C) stress, *E. coli* had the longest *D‐*value at 55°C (19.91 min) and *Listeria* at both 60°C (1.84 min) and 65°C (0.67 min). *Staphylococcus* had the lowest *D‐*values at both 55°C (9.45 min) and 60°C (1.36 min) and *E. coli* at 65°C (0.14 min). The *z‐*value was the highest for *Listeria* (7.26°C) and the lowest for *E. coli* (4.67°C).

**Table 6:** Summary of *D*‐ (min) and *z*‐values (°C) of *E. coli* (EC)*, Listeria* (LM)*, Salmonella* (SM)*,* and *Staphylococcus* (SA) that incubated during overnight growth (20 ± 2 hr) at four temperatures (15, 25, 35, and 45°C) and subsequentially subjected to thermal treatment at 55, 60, and 65°C

Thermal treatment (°C)	*D*‐ and *z*‐value of bacteria/incubation temperature (°C)															
	15				25				35				45			
	EC	LM	SM	SA	EC	LM	SM	SA	EC	LM	SM	SA	EC	LM	SM	SA
55	7.21bc	8.50b	5.82c	11.23a	31.91a	10.94b	5.29c	12.04b	25.77a	11.63b	8.71c	13.42b	19.91a	12.39b	18.59a	9.45b
60	0.62c	0.61c	0.86b	1.41a	1.36a	1.18a	0.70b	1.51a	1.20b	0.79c	0.94bc	1.57a	1.61a	1.84a	1.47a	1.36a
65	0.07b	0.11b	0.12b	0.31a	0.15b	0.17b	0.16b	0.35a	0.61a	0.45a	0.27b	0.22b	0.14b	0.67a	0.28b	0.32b
z	5.03c	5.29bc	5.85ab	6.19a	4.27b	5.38ab	6.73a	6.14a	6.13bc	7.10a	6.64ab	5.63c	4.67b	7.26a	5.47b	6.77a

Note

Means followed by the same lowercase letters in the same row within each incubation temperature are not significantly different (p > .05); detail values with standard errors can be found in appropriate tables from 2 to 5.

Overall, when all thermal stresses from 15 to 45°C were combined, *E. coli*, *Staphylococcus*, and *Listeria* demonstrated the longest thermal death decimal reduction time at 55, 60, and 65°C, respectively, while *Salmonella* required the highest thermal death decimal reduction temperatures. Of the tested bacterial species, *E. coli* generally demonstrated longer *D‐*values and lower *z‐*values indicating that the bacteria required longer time yet lower temperature increase to be destroyed as the same level as other bacterial species. Therefore, population reduction in *E. coli* associated with temperature increase alone may not be a representative indicator for the safety of food processing. In other words, because its resilience to the increase in temperatures was the shortest, simply increasing temperature alone may not assure the effective destruction of other foodborne pathogens that might have longer resilience to temperature increase.

As stated by Stringer, George, and Peck (2000), development of thermal injury, as well as inactivation, may vary from strain to strain, possibly due to environmental stresses. Findings from the present study using bacterial species associated with the vast majority of foodborne illnesses, hospitalizations, and deaths in the United States under concurrent conditions confirm that the stress response of bacteria to temperature during bacterial enumeration affects thermal tolerance of bacteria during subsequent thermal treatments. This affirms that bacterial adaptation to certain stresses may reduce the effectiveness of preservation hurdles applied during later stages of food processing and storage (Benito, Ventoura, Casadei, Robinson, & Mackey, 1999; Lou & Yousef, 1997; Oh et al., 2009; Wiegand, Ingham, & Ingham, 2009). Additionally, studies (Leenanon & Drake, 2001; Lisle et al., 1998; Swientek, 2017) reported that response to stress may not only enable survival of bacteria under more severe conditions, but also enhance their resistance during subsequent processing conditions and increase pathogenicity.

## 4 CONCLUSION

In conclusion, findings in this study clearly indicate that storage and holding temperatures similar to those encountered in food service influence the ability of foodborne pathogens to survive subsequent thermal treatments. Therefore, further research on food matrix associated with a variety of food processing related stresses (i.e., acid, fat, protein, starch, sugar, and water) as a bacterial growth and inactivation model in vitro and in situ is needed to validate current findings. Additional research on the influence of growth temperature at the same physiological stage (i.e., lag, log, and stationary phase) of cells on their sensitivity to sublethal stresses will also manifest the determination of adaptive responses in bacteria. Our findings here with further validation may also assist the food industry with the establishment of critical limits for the safe thermal treatment of food products.

## CONFLICT OF INTEREST

The authors declare no conflict of interests.

## ETHICAL STATEMENT

This study does not involve any human or animal testing.
